# Effects of Treatment Methods on the Formation, Structure, and Functional Properties of Soy Protein Amyloid Fibrils

**DOI:** 10.3390/foods14223835

**Published:** 2025-11-09

**Authors:** Qian Zhang, Yanmei Deng, Yanling Lu, Long Han, Qian Ma, Lei Guo, Fangyu Fan

**Affiliations:** 1 College of Biological and Food Engineering, Southwest Forestry University, Kunming 650224, China; 13619690455@163.com (Q.Z.); 14736649465@163.com (Y.D.); luyanling20220222@163.com (Y.L.); longhan24yn@163.com (L.H.); maqian0224@163.com (Q.M.); guolei@swfu.edu.cn (L.G.); 2 Forest Resources Exploitation and Utilization Engineering Research Center for Grand Health of Yunnan Provincial Universities, Southwest Forestry University, Kunming 650224, China; 3 Key Laboratory of Forest Disaster Warning and Control of Yunnan Province, Southwest Forestry University, Kunming 650224, Chinas

**Keywords:** soy protein isolate, amyloid fibrils, protein modification, treatment methods, structure and functional properties, food functionality

## Abstract

To investigate the effects of different treatment methods on soybean protein amyloid fibrils (SPAF), this study examined the effects of ultrasonication, cold plasma, heat, and NaCl treatment on the formation, structure, and functional properties of SPAF. SPAF structural analyses indicated that all treatments promoted SPAF assembly, with the order of effectiveness being: heat treatment > ultrasonication > cold plasma treatment > NaCl treatment. Regarding functional properties, the heat treatment group also demonstrated superior overall performance, including the highest solubility (88.15%), optimal emulsifying activity (79.63 m^2^/g) and foaming capacity (169.44%), and the highest thermal denaturation temperature (107.49 °C). Conversely, ultrasonication and cold plasma treatments, which generated shorter fibrils, offered moderate functional improvements. In contrast, NaCl treatment provided limited functional enhancement due to the formation of coarse aggregates. Consequently, heat treatment was identified as the most effective approach to promote SPAF formation and enhance functional properties. These findings provide a theoretical basis for the process optimization of SPAF in the food industry.

## 1. Introduction

Soy protein isolate (SPI), a major component of defatted soybean meal, is extensively available, cost-effective, and non-toxic. It is rich in nutritional value, with an amino acid composition that meets human nutritional requirements, and exhibits higher digestibility than most plant proteins [1]. In addition, SPI contains various bioactive components, including bioactive peptides and isoflavones. These compounds have demonstrated protective and therapeutic effects against multiple chronic metabolic diseases [2]. Nevertheless, the functionality of SPI is constrained by its molecular structure. SPI primarily contains two storage protein components, 7S (β-conglycinin) and 11S (glycinin), whose native globular structures are stabilized by hydrogen bonding and disulfide linkages. These interactions stabilize a compact spherical conformation, resulting in complex tertiary and quaternary conformations, which contribute to the poor SPI flexibility and interface properties, as well as a large apparent molecular size [3]. This limits the application of SPI in food systems. Thus, modifications are commonly applied to SPI to improve its functional properties. Among various modification methods, amyloid fibrils have attracted considerable attention because of their biocompatibility and safety, their capacity to markedly enhance protein functional properties, and their broad application potential in the food industry [4]. Amyloid fibrils are a special class of protein aggregate [5]. This fibrillar structure, characterized by its high aspect ratio, exceptional flexibility, and intertwined network, offers unique advantages for food applications owing to its biocompatibility and harmless nature [6]. Amyloid fibrils have been demonstrated to enhance both the stability and delivery efficiency of bioactive molecules [7]. Chen et al. [8] demonstrated that whey protein isolate amyloid fibrils can bind β-carotene. This not only enhances β-carotene’s water solubility and stability but also enables controlled release during in vitro gastrointestinal digestion. Ji et al. [9] reported that curcumin binds to soybean protein amyloid fibrils (SPAF), enhancing curcumin’s stability, antioxidant capacity, and sustained-release properties. In addition to being excellent carriers for bioactive molecules, amyloid fibrils can enhance protein functional properties. Liu et al. [10] demonstrated that the emulsifying properties of ovalbumin amyloid fibrils are enhanced substantially compared to native ovalbumin. Similarly, Xie et al. [11] also confirmed that the emulsifying activity index (increased from 11.72 to 19.48 m^2^/g) and foaming capacity (increased from 85% to 128%) of the fava bean protein amyloid fibrils were substantially enhanced compared to natural fava bean protein. Moreover, different treatment methods yield amyloid fibrils with diverse structure and functional properties.

Heat treatment ranks among the most prevalent techniques for protein modification. This process induces protein denaturation, aggregation, and structural rearrangement. During heat treatment, stabilizing interactions within proteins—notably noncovalent interactions (e.g., hydrogen bonding, hydrophobic and ionic interactions) and disulfide bonds—are disrupted. Subsequently, the protein undergoes ordered self-assembly by intermolecular interactions, forming amyloid fibrils [12]. Ultrasonication treatment, a non-thermal processing technology, induces cavitation, generating intense shear forces and microjets that disrupt non-covalent interactions within proteins. This phenomenon induces the conformational unfolding of proteins, exposing their hydrophobic cores and creating favorable conditions for amyloid fibril formation [13]. Cold plasma treatment, a physical modification, generates reactive oxygen and nitrogen species (RONS) along with high-energy electrons. These agents induce protein unfolding and initiate oxidative cross-linking [10]. Under specific conditions, this interaction facilitates the self-assembly of amyloid fibrils. In addition, NaCl treatment affects the assembly of amyloid fibrils primarily through electrostatic screening. This effect reduces interprotein charge repulsion, accelerates fibril aggregation, and stabilizes the fibril structure through enhanced hydrophobic interactions [14]. While studies have examined the effects of individual treatment methods on protein fibrillation, such as ultrasonication and heat treatments, systematic comparisons of heat, non-heat, physical, and chemical methods under identical conditions remain scarce.

Therefore, this study methodically analyzed the influence of four methods: ultrasonication, cold plasma, heat, and NaCl treatment on the formation, structure, and functional properties of SPAF. The extent of fibrillation and alterations in secondary structure were measured through Thioflavin T (ThT) fluorescence spectroscopy and Fourier transform infrared (FTIR) spectroscopy, respectively. The morphology of SPAF was examined using scanning electron microscopy (SEM). Furthermore, the structure under different treatments was comprehensively characterized using the surface hydrophobicity (H_0_) and X-ray diffraction (XRD). Functional properties were evaluated by determining the emulsifying activity index (EAI), emulsifying stability index (ESI), thermal stability, solubility, foaming capacity (FC), and foam stability (FS). This study systematically compared the effects of different treatments on the formation, structure, and functional properties of SPAF, aiming to provide a foundation for the high-value utilization of SPAF in the food industry.

## 2. Materials and Methods

### 2.1. Materials

The source of the soybean meal was China Grain Reserves Corporation Zhenjiang Grain and Oil Co., Ltd. (Zhenjiang, China). 1-Amino-8-naphthalenesulfonic acid (ANS) and ThT were purchased from Shanghai Yuanye Biotechnology Co., Ltd. (Shanghai, China). All chemicals were analytically pure.

### 2.2. Preparation of SPAF

SPI was acquired by alkaline solubilization and acid precipitation [15]. SPI was dispersed in deionized water at 3% (*w*/*v*). This solution was stirred for 20 min, adjusted to pH 2.0 with 1 M HCl, and centrifuged for 10 min at 8000 rpm. After collection, the supernatant was kept at 4 °C for 24 h. All treatments were conducted using the same batch of 3% (*w*/*v*) SPI dispersion at pH 2.0 as the raw material. For heat treatment, the samples were exposed to 80 °C for 10 h [16]. For ultrasonication treatment, the samples were ultrasonicated at 200 W and 25 °C for 30 min [17]. For cold plasma treatment, the samples were subjected to treatment in the reactor for 60 s at 70 V and 1.00 A [18]. For NaCl treatment, an accurately measured mass of NaCl was added to the samples based on their initial volumes to achieve a final concentration of 160 mM, followed by 20 min of magnetic stirring at room temperature [14]. All processed samples were stored at 4 °C. Samples were labeled as follows: USPAF (ultrasonication treatment), CSPAF (cold plasma treatment), HSPAF (heat treatment), and NSPAF (NaCl treatment).

### 2.3. Characteristics of SPAF

#### 2.3.1. FTIR

The freeze-dried sample was ground with KBr (1:100, m/m) and pressed into an infrared-transparent pellet. Subsequently, FTIR spectra were recorded on an FTIR spectrometer (IRPrestige-21, Shimadzu, Kyoto, Japan) from 400 to 4000 cm^−1^ at 4 cm^−1^ resolution with 16 scans. OMNIC 9.2 and Peakfit 4.12 were used to process data and calculate protein secondary structure [19].

#### 2.3.2. ThT Fluorescence

A stock solution was prepared by mixing ThT with phosphate-buffered salt solution (PBS, 150 mM NaCl, pH 7.0) at a 4:5 (*w*/*v*) ratio and sterilized by filtration through a 0.22 μm membrane. A 40 μL aliquot of the sample (10 mg/mL) was combined with 5 mL of the stock solution that had been diluted 50-fold with PBS and was incubated for 2 min at 25 °C. Fluorescence intensity was measured with excitation at 460 nm and emission at 490 nm (slit widths 5 nm for both) using a fluorescence microplate reader (Molecular Devices, San Jose, CA, USA) [14].

#### 2.3.3. Fluorescence Spectroscopy

The sample solutions were prepared at 0.06 mg/mL using deionized water that was acidified to pH 2.0 with 1 M HCl. Fluorescence spectra were acquired using a fluorescence microplate reader at an excitation wavelength of 290 nm (5 nm slit width) and by scanning the emission from 300 to 400 nm [20].

#### 2.3.4. SEM

The freeze-dried sample was analyzed using SEM (Mira LMS, Tescan Orsay Holding, Brno, Czech Republic) after gold sputtering, at 200 times magnification and an acceleration voltage of 5 kV [21].

#### 2.3.5. H_0_

A series of sample dilutions (0.1–0.5 mg/mL) was prepared in deionized water acidified to pH 2.0 using 1 M HCl. Following the addition of 50 μL of 8 mM ANS to 2 mL of the sample, the solution was incubated for 2 min in the dark. Fluorescence intensity was measured using a fluorescence microplate reader at excitation 370 nm and emission 470 nm (both slit widths 5 nm) [9].

#### 2.3.6. XRD

The XRD pattern was acquired with an Ultima IV X-ray diffractometer (Rigaku, Tokyo, Japan) using Cu-Kα radiation (λ = 1.5418 Å). The data were gathered under operating conditions of 40 kV and 40 mA over a 2θ range of 4–75° (step size: 0.013°; scanning rate: 0.056°/s) [15].

#### 2.3.7. Dityrosine

The sample solutions were prepared at 1.0 mg/mL with deionized water acidified to pH 2.0 using 1 M HCl. Fluorescence intensity was measured using a fluorescence microplate reader at excitation 325 nm and emission 420 nm (slit widths 5 nm for both) [10].

#### 2.3.8. Thermal Stability

Thermal stability was analyzed using Differential Scanning Calorimetry (DSC, 3500 Sirius, Netzsch, Selb, Germany). Samples (5.0 mg) were loaded into aluminum crucibles and heated from 30 to 200 °C at 10 °C/min under an N_2_ flow of 20 mL/min [22].

#### 2.3.9. Solubility

The sample solutions were prepared at 10.0 mg/mL with deionized water acidified to pH 2.0 using 1 M HCl. The solutions were then centrifuged for 10 min at 8000 rpm. Subsequently, the supernatant was analyzed for protein solubility using an automated Kjeldahl nitrogen analyzer (K1100, Hanon, Beijing, China).

#### 2.3.10. Turbidity

The sample solutions were prepared at 10.0 mg/mL in deionized water acidified to pH 2.0 with 1 M HCl. Absorbance was measured at 500 nm using a spectrophotometer (UV-2600, Shimadzu, Kyoto, Japan).

#### 2.3.11. Free Sulfhydryl Groups (Free-SH), Total Sulfhydryl (Total-SH), and Disulfide Bonds (S–S)

Free-SH, Total-SH, and S–S contents were measured according to the method of Liu et al. [10], with minor modifications:

Free-SH: 0.5 mL of sample was mixed with 0.02 mL of Ellman’s reagent and 2.0 mL of an 8 M urea in Tris-glycine buffer. After being vortexed for 1 min, the mixture was incubated in the dark for 30 min. The UV-2600 spectrophotometer was employed to determine the absorbance at a wavelength of 412 nm.

Total-SH: 0.5 mL of sample was mixed with 1 mL of 6 M guanidine hydrochloride and 0.025 mL of β-mercaptoethanol (β-Me), followed by incubation for 1 h. The mixture was combined with 2.5 mL of 12% (*w*/*v*) trichloroacetic acid (TCA) solution and left to stand for 1 h. The precipitate was collected by centrifugation (10,000 rpm, 10 min), washed with 12% (*w*/*v*) TCA, and centrifuged again under identical conditions, repeating this process three times to remove β-Me. The final pellet was redissolved in 2.0 mL of a solution of 8 M urea in Tris-glycine buffer, and the subsequent steps were performed following the procedure for Free-SH determination. Equation (1) was used to determine the concentrations of Free-SH (μmol/g) and Total-SH (μmol/g), and Equation (2) was used to assess the S–S (μmol/g) content:

(1) $$\mathrm{SH}(\mu \mathrm{mol}/g)=\frac{73.53\times A_{412}\times D}{C}$$

(2) $$S–S(\mu \mathrm{mol}/g)=\frac{{\mathrm{SH}}_{\mathrm{total}}-{\mathrm{SH}}_{\mathrm{free}\;\mathrm{sulfhydryl}}}{2}$$

C represents protein concentration (mg/mL), D denotes the dilution factor, and 73.53 is derived from 10^6^/(1.36 × 10^4^).

#### 2.3.12. EAI and ESI

The sample solutions were prepared at 10.0 mg/mL with deionized water acidified to pH 2.0 using 1 M HCl. Then, the sample solutions were centrifuged (8000 rpm, 10 min), after which the supernatant (15 mL) and soybean oil (5 mL) were mixed. Next, the mixture was emulsified with a high-speed disperser (FJ200-SH, Lu xi, Liaocheng, China) for 1 min at 10,000 rpm. Immediately after emulsification, a 50 μL aliquot of the emulsion was mixed with 10 mL of 0.1% (*w*/*v*) SDS solution. The absorbance at 500 nm (A_0_) was determined with a UV-2600 spectrophotometer. After 10 min, a further 50 μL of the aliquot was mixed with 10 mL of a 0.1% (*w*/*v*) SDS solution, and the absorbance at 500 nm (A_10_) was determined again using the UV-2600 spectrophotometer [23]. Equations (3) and (4) were used to calculate the EAI and ESI, respectively:

(3) $$\mathrm{EAI}(m^{2}/g)=\frac{2\times 2.303\times A_{0}\times N}{C\times \left(1-\theta \right)\times 10^{4}}$$

(4) $$\mathrm{ESI}(\%)=\frac{A_{0}}{A_{0}-A_{10}}$$

where N denotes the dilution factor, θ represents the oil volume fraction (%), C is the solution concentration (g/mL), and A_0_ and A_10_ signify the absorbance at 0 min and 10 min.

#### 2.3.13. FC and FS

The sample solutions were prepared at 10.0 mg/mL with deionized water acidified to pH 2.0 using 1 M HCl. The sample (V_1_) was homogenized for 1 min at 10,000 rpm using a high-speed disperser. The volume immediately after homogenization was recorded as V_2_. After standing for 10 min, the volume was recorded as V_3_. Equations (5) and (6) were used to calculate the FC and FS, respectively:

(5) $$\mathrm{FC}(\%)=\frac{V_{2}-V_{1}}{V_{1}}\times 100\%$$

(6) $$\mathrm{FS}(\%)=\frac{V_{3}-V_{1}}{V_{1}}\times 100\%$$

V_1_, V_2_, and V_3_ are the sample volumes before homogenization, immediately after homogenization, and after standing for 10 min, respectively.

### 2.4. Statistical Analysis

Data were presented as the mean ± standard deviation (SD) of three independent experiments (*n* = 3). Data were analyzed using IBM SPSS Statistics 27. Specifically, one-way ANOVA followed by Waller–Duncan post hoc tests was applied to compare groups. Graphs were generated using Origin 2021 software. Statistical significance is defined as *p* < 0.05.

## 3. Results and Discussion

### 3.1. FTIR Analysis

**Figure 1:**
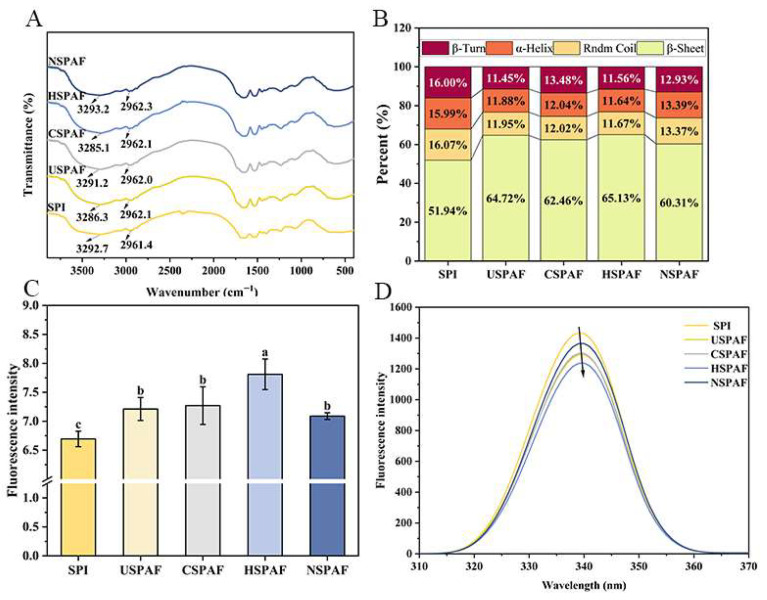
FTIR spectra (**A**), quantitative secondary structure analysis (**B**), ThT fluorescence intensity (**C**), and intrinsic fluorescence spectra (**D**) of SPI, USPAF, CSPAF, HSPAF, and NSPAF. Data in (**C**) is presented as mean ± SD (n = 3). Different lowercase letters above the bars indicate significant differences (*p* < 0.05).

FTIR can assess changes in protein functional groups and chemical bonds. Figure 1A shows that the 3000–3500 cm^−1^ range peak is associated with O-H and N-H stretching vibrations, with peak shifts indicating altered hydrogen-bonding interactions [24]. Notably, the redshift observed for NSPAF implies the formation of new hydrogen bonds during fibrillation. In contrast, the blueshift in USPAF, CSPAF, and HSPAF indicates that the extent of hydrogen bond destruction during fibrillation outweighed the formation of intermolecular hydrogen bonds, which is consistent with Mo et al.’s observations during whey protein fibrillation [25]. Additionally, the peak at 2960 cm^−1^, which can be used to analyze hydrophobic interactions, is attributed to C-H stretching vibrations in CH_3_ and CH_2_ groups [26]. Compared to SPI, the redshift in all samples reflects increased hydrophobicity induced by hydrolysis and fibrillation [27]. Given the heightened sensitivity of the amide I region (1600–1700 cm^−1^) to secondary structure perturbations, it is widely employed to monitor protein conformational transitions [28].

As shown in Figure 1B, the β-sheet content for SPI, USPAF, CSPAF, HSPAF, and NSPAF was 51.94%, 64.72%, 62.46%, 65.13%, and 60.31%, respectively. Following fibrillation, all four treatments exhibited an increase in the β-sheet content compared to SPI. These results are in line with the findings of Oboroceanu et al. [29], who through peak area fitting of the amide I region, confirmed that the formation of the β-sheet structure is a key characteristic of the fibrillation process. Among these, HSPAF exhibited the highest β-sheet content, with 65.13%, owing to the 10 h heat treatment, providing sustained energy to unfold the protein structure. Then, loose peptide chains undergo directional arrangement, ultimately forming a regular structure dominated by β-sheets [21]. Feng et al. [30] showed that the formation of protein amyloid fibrils is essentially a molecular self-assembly process. The increase in the β-sheet content of USPAF is primarily due to cavitation caused by ultrasonication, which breaks non-covalent bonds in proteins, resulting in structural changes that promote amyloid fibril formation [31]. The main reason for the increase in CSPAF β-sheet content is that cold plasma treatment generates energetic particles, UV radiation, and RONS, such as ozone and peroxides. These reactive agents induce protein unfolding and initiate oxidative cross-linking, thereby driving amyloid fibrillation [18]. An increase in NSPAF β-sheet content was observed, possibly due to NaCl-induced modulation of protein charge distribution and intermolecular interactions. This modulation promotes amyloid fibril formation. Notably, the treatments could be ranked in the following order based on their effectiveness in promoting β-sheet formation: heat > ultrasonication > cold plasma > NaCl. Thus, heat treatment most effectively induced β-sheet formation and amyloid fibril self-assembly.

### 3.2. ThT Fluorescence Analysis

ThT fluorescence intensity is widely used to monitor amyloid fibril formation. ThT dye specifically recognizes and intercalates into the β-sheets, exhibiting fluorescence enhancement that correlates with the content of protein amyloid fibrils [15]. Figure 1C illustrates that the fluorescence intensities of SPI, USPAF, CSPAF, HSPAF, and NSPAF were 6.69, 7.21, 7.27, 7.81, and 7.09 a.u., respectively. All treated samples exhibited significantly higher fluorescence intensities than SPI (*p* < 0.05). These results confirm the formation of amyloid fibrils featuring β-sheets in the samples [32]. The low fluorescence intensity of SPI may be due to the absence of extensive cross-β amyloid structures in its native state. HSPAF exhibited the highest fluorescence intensity, likely due to its degree of fibrillation and greatest β-sheet content [33]. The results indicate that the effectiveness of treatments in promoting SPAF formation follows the order: heat treatment > ultrasonication treatment > cold plasma treatment > NaCl treatment. Among them, heat treatment is the most effective in promoting amyloid fibril formation.

### 3.3. Fluorescence Spectroscopy Analysis

Shifts in the fluorescence spectrum are a result of altered polarity around tyrosine (Tyr) and tryptophan (Trp) residues. Consequently, perturbations to protein tertiary structure can be monitored by observing these concomitant spectral shifts [34]. The hydrophobic regions of SPI contain Trp and Tyr, which promote β-sheet formation and facilitate π-π stacking interactions [31]. Figure 1D illustrates that, compared with SPI, USPAF, CSPAF, HSPAF, and NSPAF all exhibited reduced maximum fluorescence intensities. This decrease may be attributed to protein hydrolysis, molecular rearrangement, and encapsulation of Trp and Tyr within the newly formed protein aggregates. HSPAF exhibited the lowest fluorescence intensity among all samples, likely due to its highest degree of fibrillation (as evidenced by ThT fluorescence), which leads to greater burial of Trp residues. Additionally, Xia et al. [20] found that Trp residues exhibit sensitivity to heat-induced oxidative effects, resulting in lowered fluorescence emission intensity. The maximum emission wavelengths of USPAF, CSPAF, HSPAF, and NSPAF exhibited red shifts in the fluorescence spectra. This red shift indicates that the microenvironment around the Trp and Tyr residues becomes more polar. These indicate that, during the fibrillation process, SPI undergoes structural changes involving partial unfolding/disaggregation and self-assembly. According to Zhang et al. [35], during fibrillation, the fluorescence of whey protein amyloid fibrils decreased and underwent a red shift over time.

### 3.4. SEM Analysis

**Figure 2:**
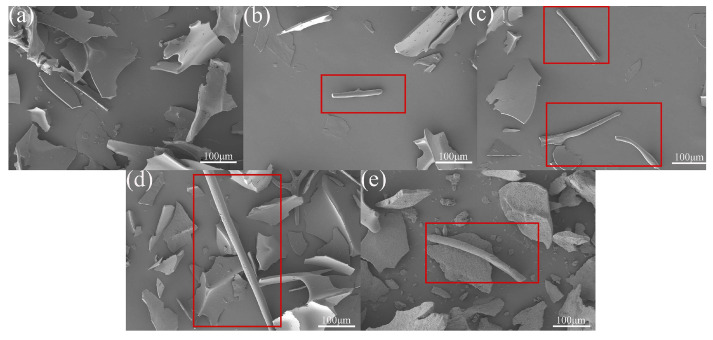
SEM of SPI (**a**), USPAF (**b**), CSPAF (**c**), HSPAF (**d**), and NSPAF (**e**). The red squares highlight aggregation structures.

SEM was employed to characterize the morphology and macroscopic aggregate structure of the freeze-dried samples. In Figure 2, HSPAF exhibits continuous, well-defined aggregates, attributable to the 10 h heat treatment, which induces protein unfolding and promotes the ordered self-assembly of β-sheets, forming a highly ordered structure [21]. In addition, the enhanced cross-linking of intermolecular disulfide bonds during the heat treatment process further promotes the longitudinal extension of aggregates [36]. USPAF exhibited short aggregates, likely due to localized high pressure and microjets generated by ultrasonic cavitation, which not only induces protein fragmentation but also disrupts noncovalent bonds [37]. CSPAF exhibited short aggregates, likely because cold plasma-active species cause random chemical modifications of proteins [38]. NSPAF showed the highest surface roughness. This can be explained by Na^+^, a weak salting-out ion, which partially disrupts the protein hydration layer and, via electrostatic screening, weakens intermolecular repulsion, thereby enhancing hydrophobic interactions and promoting localized aggregation [39]. An excessively high aggregation rate can lead to non-uniform aggregates. In summary, the treatments yielded distinct aggregate morphologies, reflecting their different mechanisms of action.

### 3.5. H_0_ Analysis

**Figure 3:**
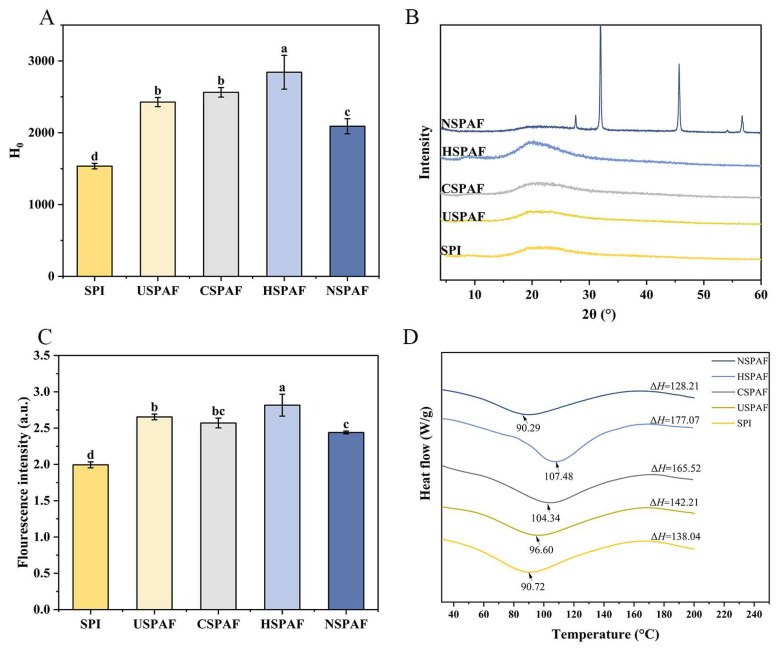
H_0_ (**A**), XRD spectra (**B**), dityrosine content (**C**), and DSC thermograms (**D**) of SPI, USPAF, CSPAF, HSPAF, and NSPAF. Data in (**A**,**C**) are presented as mean ± SD (n = 3). Different lowercase letters above the bars indicate significant differences (*p* < 0.05).

ANS specifically binds to exposed hydrophobic protein surfaces. When proteins undergo conformational changes or denaturation, buried hydrophobic amino acid residues become solvent-exposed. The binding of ANS to these exposed residues leads to an increase in fluorescence intensity [40]. Figure 3A illustrates that the H_0_ values of USPAF, CSPAF, HSPAF, and NSPAF were significantly increased compared to those of SPI (*p* < 0.05). During protein amyloid fibril formation, proteins are hydrolyzed into low-molecular-weight peptides, causing the hydrophobic groups within the natural proteins to become exposed, increasing H_0_ [41]. Among them, the highest H_0_ in HSPAF could be attributed to the heat treatment (80 °C, 10 h), which provides a continuous and uniform energy input, resulting in protein denaturation and revealing additional hydrophobic groups. These revealed hydrophobic groups promote protein aggregation. It was reported by Green et al. [42] that, during fibrillation, hydrophobic interactions promote the formation of β-sheets. The differences in H_0_ values reflect the varying contributions of hydrophobic interactions during protein fibrillation. The highest H_0_ value was found in the heat treatment, suggesting that hydrophobic interactions contribute to the fibrillation process under this condition.

### 3.6. XRD Analysis

XRD is employed for crystal structure analysis [43]. By analyzing the displacement of diffraction angles (2θ) and intensity alterations in diffraction patterns, the structural orderliness of molecular arrangements in protein aggregates can be evaluated [44]. Figure 3B shows the XRD patterns of the samples. All samples showed characteristic diffraction peaks of β-sheets at 2θ = 18.9° [15]. Compared to SPI, peak intensities were enhanced in USPAF, CSPAF, and HSPAF, indicating that these treatments effectively promoted the formation of β-sheet. Among them, HSPAF exhibited the most pronounced diffraction enhancement effect, consistent with the degree of fibrillation indicated by ThT fluorescence and the secondary-structure results from FTIR. This is attributed to the highest degree of fibrillation and the highest β-sheet content in HSPAF [16]. However, the characteristic peak intensity at 2θ = 18.9° in NSPAF was weakened. This may be attributed to Na^+^-induced protein aggregation, which leads to the formation of partially irregular structures [45]. Additionally, the sharp peaks observed in the XRD spectrum after NaCl treatment were attributed to the overlap between the crystalline diffraction peaks of NaCl and the characteristic peaks of protein amyloid fibrils, according to the ICDD standards (PDF 00-005-0628) [46].

### 3.7. Dityrosine Analysis

Dityrosine is integral to the stability and architecture of amyloid fibrils and exhibits fluorescent properties with a maximum emission wavelength of 420 nm [47]. In Figure 3C, the dityrosine contents of SPI, USPAF, CSPAF, HSPAF, and NSPAF were 1.99, 2.65, 2.57, 2.81, and 2.44 a.u., respectively. The dityrosine levels in USPAF, CSPAF, HSPAF, and NSPAF were higher than those of SPI, likely owing to oxidative cross-linking of tyrosine residues during fibrillation. When SPI undergoes depolymerization and reassembly, the tyrosine residues in its structure become exposed from the hydrophobic core, making them susceptible to oxidation to form dityrosine [10]. Among them, HSPAF had the highest dityrosine content, probably because the heat treatment lasting 10 h drives tyrosine residues, originally concealed within the hydrophobic core, into exposure to a polar microenvironment. This subsequently triggers oxidative modification to form dityrosine. The results indicate that when protein self-assembles to form amyloid fibrils, dityrosine cross-linking also occurs. Liu et al. [10] obtained similar results, demonstrating that the driving force behind ovalbumin fibrillation involves not only hydrogen bonds and hydrophobic interactions but also dityrosine covalent bonds.

### 3.8. Thermal Stability Analysis

The endothermic peak temperature in the DSC curve corresponds to the denaturation temperature (T_d_) of proteins. The enthalpy change (ΔH) reflects the energy required for protein denaturation [48]. Figure 3D shows that T_d_ and ΔH of SPI were 90.72 °C and 138.05 J/g, respectively. The T_d_ values of USPAF, CSPAF, and HSPAF increased to 96.59 °C, 104.33 °C, and 107.49 °C, respectively, with their corresponding ΔH also rising to 142.21 J/g, 165.52 J/g, and 177.07 J/g. Among these, HSPAF showed the highest thermal stability. This can be attributed to a two-step mechanism: first, the structural unfolding induced by pre-denaturation with heat, which exposes buried hydrophobic regions and active sites; second, the subsequent continuous heating that provides the necessary energy for the self-assembly of peptide chains into well-ordered amyloid fibrils dominated by β-sheet structures, thereby forming more amyloid fibrils [16]. NSPAF exhibited lower T_d_ and ΔH, at 90.29 °C and 128.21 J/g, respectively, than SPI. This reduction is likely due to NaCl treatment disrupting electrostatic repulsion among proteins, which promotes protein refolding and the formation of partially disordered aggregates, reducing thermal stability. The increase in disordered structures typically leads to a lower ΔH, which is consistent with the findings of Jiang et al. [48]. Combined analysis of fluorescence spectroscopy and secondary structure content demonstrated that the treatment groups with enhanced thermal stability (including heat, cold plasma, and ultrasound) consistently exhibited higher β-sheet content and lower tryptophan fluorescence intensity compared to those of SPI. This suggests that the ordered assembly of β-sheet enriched amyloid fibrils drives enhanced thermal stability. The enhanced thermal stability resulting from protein fibrillation aligns with earlier observations by Bolder et al. [49].

### 3.9. Solubility Analysis

**Figure 4:**
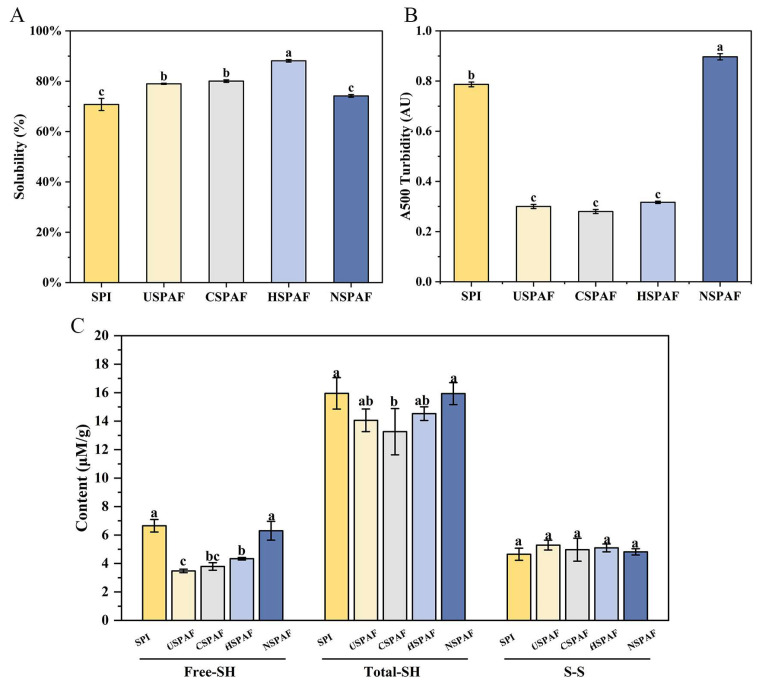
Solubility (**A**), turbidity (**B**), and contents of Free-SH, Total-SH, and S–S of SPI, USPAF, CSPAF, HSPAF, and NSPAF. Data in (**A**–**C**) are presented as mean ± SD (n = 3). Different lowercase letters above the bars indicate significant differences (*p* < 0.05).

Protein solubility affects numerous key functional properties, such as emulsification, gelation, and foaming [50]. Figure 4A shows that the solubility of SPI was 70.79%. After treatments, the solubilities of USPAF, CSPAF, HSPAF, and NSPAF were 78.99%, 80.05%, 88.13%, and 74.19%, respectively. USPAF, CSPAF, and HSPAF experienced a significant enhancement in solubility (*p* < 0.05). During fibrillation, this increase is due to structural changes that expose buried hydrophilic amino acid residues, as well as hydrolysis, which produces low-molecular-weight soluble peptides. These changes collectively enhance solubility. The hydrolysis products then undergo ordered self-assembly via hydrophobic interactions and β-sheet formation, yielding SPAF with excellent surface characteristics. Moreover, during fibrillation, protein hydrolysis and self-assembly maintain a dynamic equilibrium. This balance ensures SPAF formation without excessive aggregation, further increasing sample solubility [48]. Among all samples, HSPAF exhibited the highest solubility, likely due to the formation of well-structured amyloid fibrils through heat-induced protein self-assembly. Wang et al. [16] demonstrated that proteins heated for 8–10 h at pH 2.0 and 85 °C develop an optimal fibrous structure. Nevertheless, the solubility values of SPI and NSPAF were found to be virtually identical, with no significant difference (*p* > 0.05). This result is likely due to the ionic effect during fibrillation, and Na^+^ may accelerate aggregation, thereby disrupting the dynamic equilibrium maintained between proteolysis and self-assembly.

### 3.10. Turbidity Analysis

Turbidity is directly related to the aggregation state and particle size of the sample and thus can indirectly reflect solubility [51]. Figure 4B illustrates that the turbidity of SPI was 0.78 AU, which may be due to the presence of numerous insoluble proteins. The turbidity of USPAF, CSPAF, and HSPAF decreased significantly to 0.30 AU, 0.28 AU, and 0.32 AU, respectively (*p* < 0.05), compared with SPI. The decrease is likely due to the mechanical effect of ultrasound, modification of proteins by reactive oxygen species in plasma, and thermotropic denaturation, respectively. These treatments induce the expansion and restructuring of the proteins, which promote the generation of soluble SPAF. The turbidity value of the NSPAF was 0.89 AU, likely due to NaCl-induced formation of larger protein aggregates. Ji et al. [45] also demonstrated that samples with 160 mM NaCl exhibited larger particle sizes. This finding is consistent with the results regarding solubility.

### 3.11. Free-SH, Total-SH, and S–S Analysis

During amyloid fibril formation, protein rearrangements lead to the exposure of buried sulfhydryl groups. These exposed sulfhydryl groups are consumed during self-assembly through disulfide bond formation and sulfhydryl-disulfide exchange reactions, which are critical for stabilizing the fibril structure [52]. As shown in Figure 4C, the Free-SH and Total-SH contents of SPI were 6.65 and 15.95 μmol/g, respectively. After treatments, the Free-SH contents of USPAF, CSPAF, HSPAF, and NSPAF were 3.48, 3.79, 4.34, and 6.30 μmol/g, respectively. The corresponding Total-SH contents were 14.05, 13.26, 14.53, and 15.93 μmol/g, respectively. The observed decline is due to the structural unfolding of SPI during fibrillation, which promotes the oxidation of exposed -SH groups into S–S bonds [53]. Among them, CSPAF showed the lowest Free-SH and Total-SH content, likely due to over-oxidation of cysteine residues and disulfides by RONS generated by cold plasma, converting a fraction into sulfinic and sulfonic acids [54]. The Free-SH and Total-SH contents showed no significant difference between NSPAF and SPI (*p* > 0.05), suggesting that NaCl treatment may promote SPAF formation through other mechanisms. As shown in Figure 4C, the S–S content remained statistically unchanged across all treatment groups (*p* > 0.05), indicating that it was not the primary driver of SPAF formation under these conditions.

### 3.12. FC and FS Analysis

**Figure 5:**
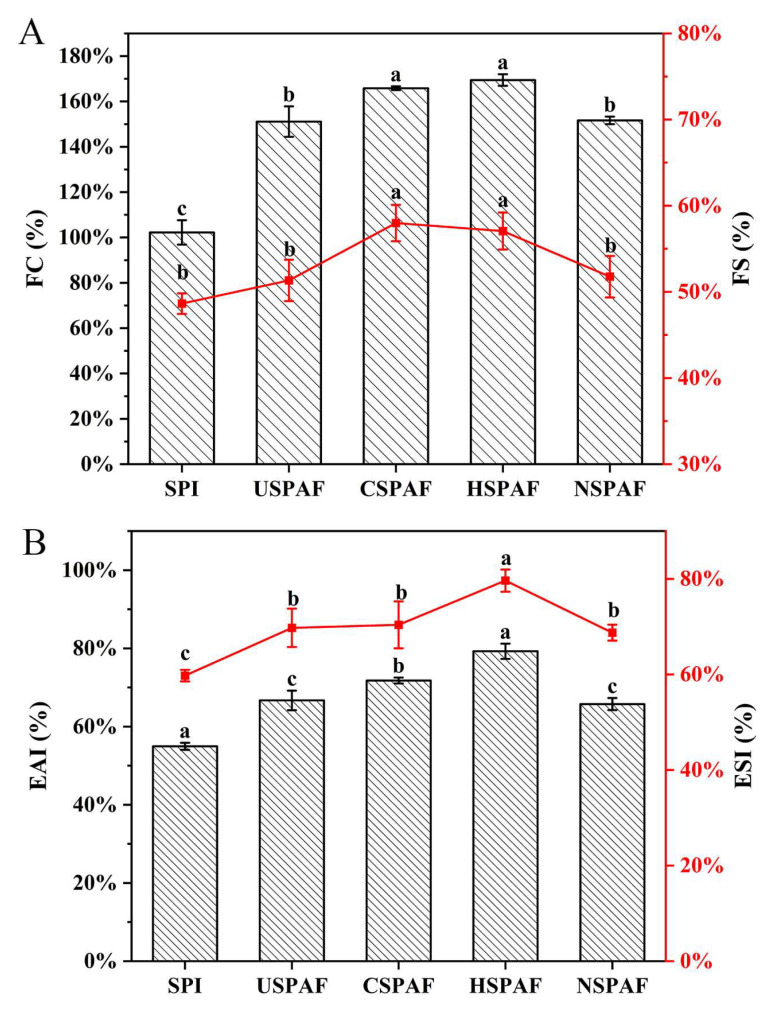
FC and FS (**A**) and EAI and ESI (**B**) of SPI, USPAF, CSPAF, HSPAF, and NSPAF (FS and ESI are shown in red). Data in (**A**,**B**) are presented as mean ± SD (n = 3). Different lowercase letters above the bars indicate significant differences (*p* < 0.05).

FC reflects the protein’s ability to rapidly adsorb at the air–water interface and generate foam upon shear. FS indicates the protein’s ability to maintain foam structure and delay foam collapse [55]. Figure 5A illustrates that the FC and FS of SPI were 102.22% and 48.64%, respectively. The FC values for USPAF, CSPAF, HSPAF, and NSPAF reached 151.11%, 165.83%, 169.44%, and 151.67%, with corresponding FS values of 51.33%, 57.99%, 57.06%, and 51.77%, respectively. The significant improvements (*p* < 0.05) in FC and FS for all treated samples (USPAF, CSPAF, HSPAF, NSPAF) compared to SPI were closely associated with the increased formation of amyloid fibrils. During fibrillation, protein unfolding exposes hydrophobic groups and enhances molecular flexibility. These changes promote more rapid and efficient formation of a viscoelastic protein film at the interface, thereby enhancing FC [56]. The FS increase results from the formation of SPAF, which enhances its adsorption capacity at the interface. This process effectively reduces interfacial tension and strengthens the viscoelasticity of the interfacial film, thereby delaying foam rupture [57]. Among all groups, HSPAF exhibited the highest FC and FS, while no significant difference was found (*p* > 0.05). Based on ThT fluorescence results, heat treatment appears to be the most effective means for inducing protein fibril formation. Subjected to a 10 h heat treatment, the sample underwent more extensive molecular cross-linking, resulting in the formation of a greater quantity of stable SPAF. Cold plasma may act on the surface of proteins through reactive species generated, increasing the surface properties of the proteins and thereby enhancing their adsorption kinetics and interfacial spreading ability at the air–water interface, ultimately resulting in improvements in FS and FC. Oboroceanu et al. [55], who also reported that protein fibrillation can improve both FC and FS, observed similar phenomena in their study.

### 3.13. EAI and ESI Analysis

EAI quantifies protein adsorption at the oil–water interface, whereas ESI assesses emulsion stability over time [58]. Figure 5B illustrates that, compared with SPI, the EAI and ESI of USPAF, CSPAF, HSPAF, and NSPAF were significantly higher (*p* < 0.05). This can be attributed to SPAF having a greater specific surface area and higher interfacial activity, which facilitates faster diffusion to the interface and the formation of stable interfacial films. Additionally, protein unfolding during fibrillation increases H_0_. The increased H_0_ enhances protein-oil interactions, leading to greater adsorption at the oil–water interface [30]. Man et al. [51] also confirmed that fibril aggregates, due to their unique surface characteristics and structure, could serve as highly promising emulsifiers in food processing. Among all samples, HSPAF showed the highest EAI (79.63 m^2^/g) and ESI (79.26%). Based on the H_0_ and ThT fluorescence analysis, this phenomenon may be because HSPAF has a higher H_0_ value than other groups. Additionally, heat induced the formation of more SPAF, which possesses superior surface properties. However, NSPAF showed the lowest EAI (65.75 m^2^/g) and ESI (68.73%) among all treated samples, likely due to the ionic environment favoring the formation of larger, irregular aggregates. The increase in these aggregates reduced the effective coverage of emulsion droplet surfaces. Therefore, the unique structural characteristics induced by different treatments impart specific functional advantages to the samples. Heat treatment induces the formation of highly ordered aggregates, and the samples exhibit optimal functional properties, making them suitable for high-stability emulsions and gels. Ultrasonication treatment produces short aggregates conducive to rapid adsorption, making them ideal for foaming systems. Cold plasma treatment enhances interfacial activity through surface modification, which is applicable in fine emulsions and composite gels. Conversely, NaCl treatment forms coarse aggregates, resulting in limited improvement in functional properties.

### 3.14. Correlation Analysis

**Figure 6:**
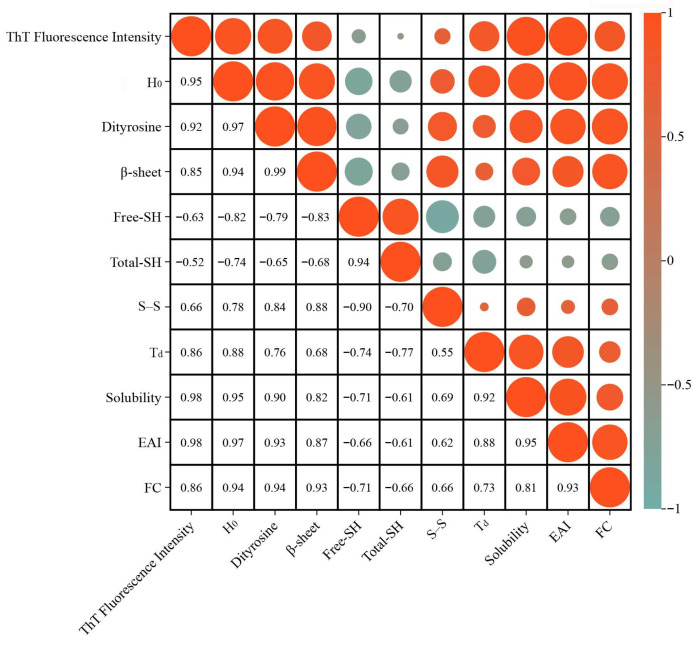
Statistical heat map correlation matrix of the relationship between different variables.

Based on Pearson correlation analysis (Figure 6), the relationship between the degree of SPAF formation and its structures and functional properties was investigated. The positive correlation between ThT fluorescence intensity and β-sheet content, H_0_, and dityrosine content confirms that SPAF formation is fundamentally an ordered self-assembly process dominated by β-sheets [30], and indicates that during fibrillation, proteins unfold and expose hydrophobic regions and tyrosine residues, which provide the key driving force for fibril assembly. In addition, the negative correlation between ThT fluorescence intensity and Free-SH and Total-SH, coupled with its positive correlation with S–S content, indicates that the oxidation of -SH to form S-S further promotes intermolecular cross-linking, thereby stabilizing the fibrillar structure [52]. Further analysis indicates that the positive correlation between ThT fluorescence intensity and T_d_, solubility, EAI, and FC demonstrates that the SPAF exhibits excellent thermal stability, solubility, and interfacial functionality. Moreover, H_0_, dityrosine content, and β-sheet content positively correlate with T_d_, solubility, EAI, and FC. This indicates that enhanced hydrophobic interactions, dityrosine formation, and orderly β-sheet dominated structural stacking act synergistically during fibrillation. Together, they promote SPAF formation, thereby driving improvements in surface activity, thermal stability, and solubility. Notably, the positive correlation between H_0_ and solubility likely stems from the protein conformational transitions and ordered assembly during fibrillation. Protein unfolding increases H_0_, while hydrolysis exposes hydrophilic groups and generates small soluble peptides. Furthermore, the ordered rearrangement of peptide chains forms structurally regular soluble fibers. In this process, the enhancement of solubility plays a dominant role, resulting in the overall observed trend of their simultaneous increase. The aforementioned conclusions are corroborated by the experimental results presented in Section 3.1, Section 3.2, Section 3.3, Section 3.4, Section 3.5, Section 3.6, Section 3.7, Section 3.8, Section 3.9, Section 3.10, Section 3.11, Section 3.12, Section 3.13 and Section 3.14 of this paper. In summary, the formation process of SPAF involves multi-level structural evolution and the synergistic interaction of multiple molecular forces, with its structural order being closely associated with enhanced functional properties.

## 4. Conclusions

This study examined the impact of ultrasonication, cold plasma, heat, and NaCl treatment on the formation, structure, and functional properties of SPAF. The results indicated that all four treatments promoted β-sheet formation and SPAF self-assembly through the major driving forces of hydrogen bonding, hydrophobic interactions, and oxidative cross-linking, with efficacy in the order: heat > ultrasonication > cold plasma > NaCl treatment. Specifically, the heat treatment group exhibited the most outstanding comprehensive performance, featuring the highest β-sheet content, the most distinct fibrillar morphology, and optimal solubility, thermal stability, and interfacial properties. Although ultrasonication and cold plasma treatments differ in their mechanisms of action, both effectively modify the interfacial behavior of proteins and exhibit comparable performance in functional properties. In contrast, NaCl treatment formed coarse aggregates, resulting in limited improvement of functional properties. Consequently, heat treatment is the most effective method for preparing high-performance SPAF, and its operation is convenient and straightforward, possessing excellent potential for industrial application. These findings provide a basis for the strategic, high-value utilization of proteins in the food industry. Future research should further investigate multimethod processing synergies and evaluate the performance of SPAF within complex food matrices, including its digestive characteristics, long-term stability, and efficacy as a novel emulsifier or encapsulation system for bioactive substances, thereby advancing its application in functional foods.

## Data Availability

The original contributions presented in this study are included in the article. Further inquiries can be directed to the corresponding author.
